# Supplementary material for: The Structural and Functional Responses of Rhizosphere Bacteria to Biodegradable Microplastics in the Presence of Biofertilizers
Source: Plants (Basel). 2024 Sep 20;13(18):2627. doi: 10.3390/plants13182627 (PMC11434756; doi:10.3390/plants13182627)
Supplement: Supplementary file 1 [file plants-13-02627-s001.zip › plants-3188069-supplementary.pdf]

Supplementary Information

Structural and functional responses of rhizosphere bacteria to biodegradable microplastics in the presence of biofertilizers

Xueyu Cheng[a,b,c], Xinyang Li[a,b,c], Zhonghua Cai[a,b,c], Zongkang Wang[d*], Jin Zhou[a,b,c**]

[a] Marine Ecology and Human Factors Assessment Technical Innovation Center of Natural Resources Ministry, Tsinghua Shenzhen International Graduate School, Shenzhen 518055, Guangdong Province, P. R. China

[b] Shenzhen Public Platform for Screening and Application of Marine Microbial Resources, Institute for Ocean Engineering, Shenzhen International Graduate School, Tsinghua University, Shenzhen 518055, Guangdong Province, P. R. China

[c] Shenzhen Key Laboratory of Advanced Technology for Marine Ecology, Institute for Ocean Engineering, Shenzhen International Graduate School, Tsinghua University, Shenzhen 518055, Guangdong Province, P. R. China

[d] Ecological Fertilizer Research Institute, Shenzhen Batian Ecological Engineering Co., Ltd., Shenzhen, Guangdong Province, P. R. China

[**]Corresponding author: Jin Zhou

E-mail address: zhou.jin@sz.tsinghua.edu.cn

Supplementary methods

Text S1. Bioorganic fertilizer were produced by the Shenzhen Batian Ecological Technique Co., Ltd. (China). The organic fertilizer containing 29.27% organic matter, 1.52% N, 1.23% P_2O_5, and 2.02% K_2O was first made from raw materials, namely urea, black sticky powder, humic acid powder, and phosphate fertilizer. Next, *Bacillus amyloliquefaciens* was fermented by adding 3×10^{10} spores per gram into the rotted organic material; then it was granulated by rotary drum and dried at room temperature to make the biofertilizer. The latter's colony number was detected as being 2×10^8 spores·g^{-1}.

Text S2. Two types of MPs particles were studied: PE MPs (CAS No.: 9002–88-4; PEM1850A, China Petrochemical Corporation, China) and Bio MPs. The industrial masterbatch of Bio MPs consisted of 70% PBAT (CAS No.: 55231–08-8; Xinjiang Lanshan Tunhe Technology Corporation TH801S, Tunhe, China) and 30% PLA (CAS No.: 26023–30-3; FY201, BBCA Biochemical Co., Ltd., China). The PE MPs and Bio MPs were both purchased from the Huachuang Chemicals Co. Ltd., Dongguan, China.

Specifically, to estimate the particle size distribution, a portion of the original sample of MPs was collected and sieved at a maximum particle size of 2000 μm. Particles smaller than 2000 μm were analyzed using a Mastersizer 2000 and a HydroEV disperser with water. The size distribution was estimated by laser diffraction, with particles of 0.02 to 2000 μm detected. All measurements were made in triplicate. The size distribution was estimated in terms of relative proportions (percentages), this determined by the diffraction angle of particles according to the equivalent sphere diameter theory and the Mie approximation via mathematical and geometrical functions. For the MPs' surface functional group composition detection in

FT-IR, the scanning range was set to 4000–475 cm^{-1}, with a scan area of 1000 × 1000 μm and a resolution of 4 cm^{-1}. The MP samples were scanned 100 times. All obtained spectra data were processed using OMNIC™ Specta software (Thermo Scientific, USA).

After 28 days of experiments, the degradation of Bio-MPs was as follows. Our study was conducted in a soil environment, where the degradation of PLA was observed to be extremely slow and therefore negligible over the 28-day experimental period (DOI:10.1111/j.1747-0765.2007.00169.x). As for the PBAT material, it is not only degradable in a composting environment. In soil environment, degradation can start after two weeks (DOI:10.1016/j.chemosphere.2007.10.074). In our previous research, we conducted a comprehensive analysis of the surface morphology, primary components, and functional groups of PBAT-MP before and after 28 days of exposure to biofertiliser-rich soil environments. We employed scanning electron microscopy, EDX spectroscopy, and Fourier transform infrared spectroscopy to investigate the changes in these properties (DOI:10.1016/j.jenvman.2024.120071).

Text S3. While removing the plants from the pots, the soil loosely adhering to their root surface was shaken off, thus laving about 1 mm of soil on the root surface as rhizosphere soil. Plant root zone samples were placed in 50 mL test tubes, each containing 30 mL of sterile phosphate-buffered saline (PBS, pH 7.0) solution, and this stirred vigorously to dislodge all rhizosphere soil from the root surface. Then the rhizosphere soil was centrifuged at 10 000 g for 30 Section (4°C) and then concentrated. After that, plant root zone samples without their rhizosphere soil were washed two more times with sterile PBS solution, to remove any visible adhering soil .

Text S4. All PCR reactions were carried out with 15 µL of Phusion® High-Fidelity PCR Master Mix (New England Biolabs, USA). To determine the accuracy of the PCR products, their assays were performed using 2% (w/v) agarose gel electrophoresis. Sequencing libraries were then generated using the NEB Next® Ultra DNA Library Preparation Kit (Illumina, USA), according to the manufacturer's recommendations, with index codes added. Library quality was assessed by the Agilent 5400 system (Agilent Technologies, USA). Finally, all libraries were sequenced on the Illumina NovaSeq platform to generate 250-bp paired-end reads. QIIME software (v1.9.1) was used to analyze the raw data obtained and to remove sequences with translation failures. Next, UPARSE software (v7.0.1) was used to cluster the remaining sequencing reads into operational taxonomic units (OTUs) with at least a 97% similarity.

Text S5. The original sequencing data underwent preprocessing using Kneaddata software to remove sequencing adapters and readouts containing low-mass bases (default quality threshold ≤20) and sequences with a final length <50 bp were removed using Trimmomatic. The clean data were screened against host genomes using Bowtie2 software to filter out host source readings and eliminate possible host contamination. FastQC was used to evaluate the effectiveness of quality control. The processed sequences were compared with the protein database (UniRef90) using HUMAnN2 software (based on DIAMOND), and the annotation information and relative abundance tables of each functional database were obtained according to the UniRef90 ID and its correspondence to each database. The putative amino acid sequences translated from the gene catalog were compared with the Kyoto Encyclopedia of Genes and Genomes (KEGG) pathways database using BLAST (version 2.2.21), with emphasis on carbon cycle genes. Gene abundance was summarized as the number of recombinase A copies detected in each metagenomic genome.

Supplementary results

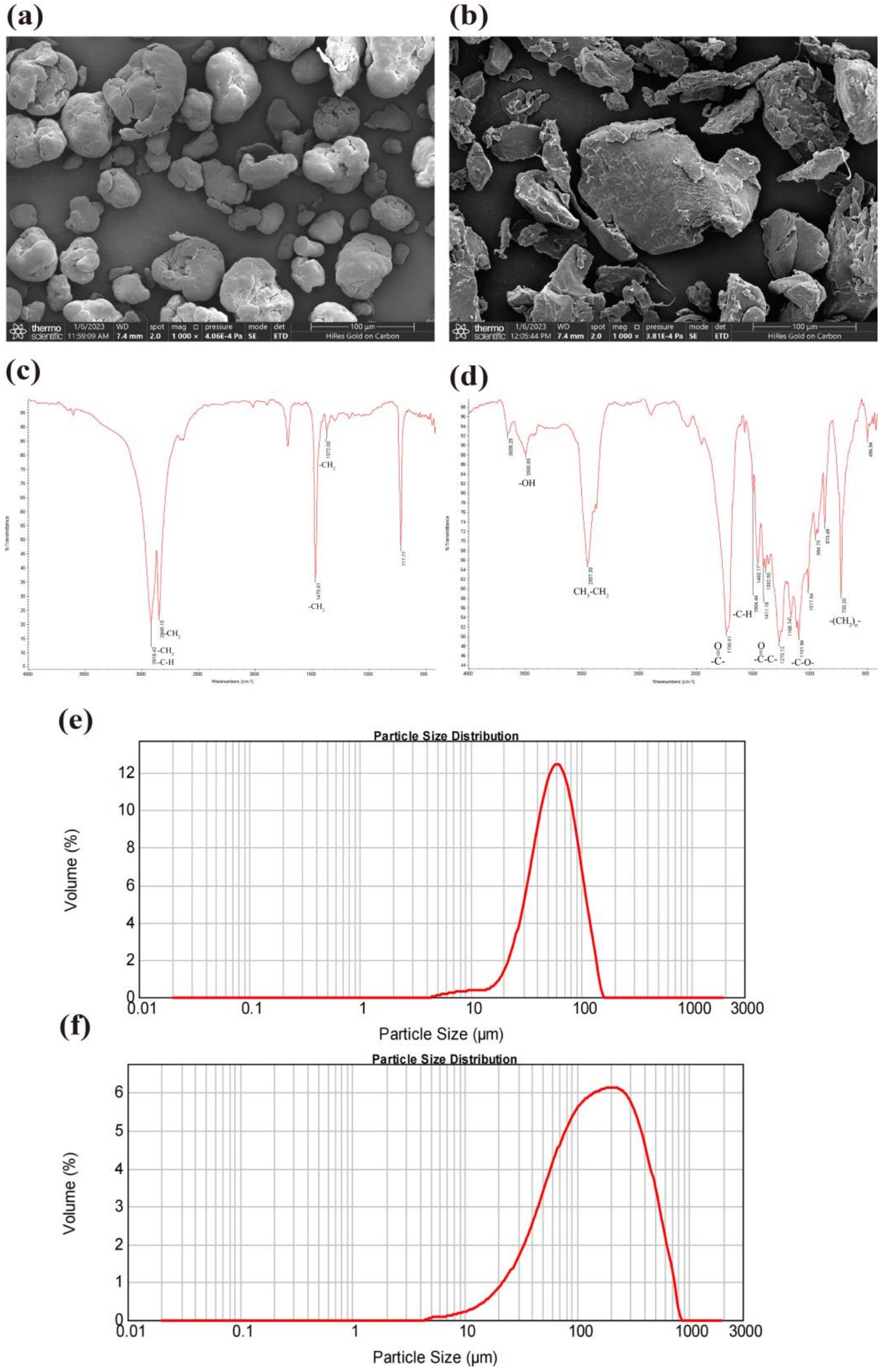

Figure S1. Scanning electron microscope, fourier transform infrared spectrometer spectra and laser particle size analyzer images for (a, c, e) PE MPs and (b, d, f) Bio MPs.

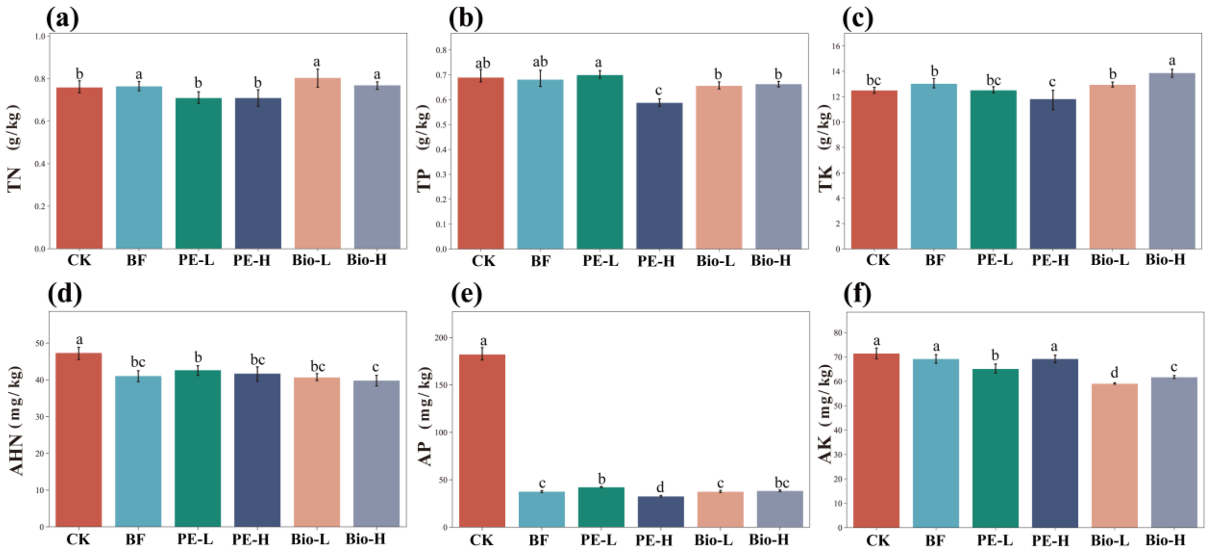

Figure S2. Effect of MPs on soil properties existence with biofertilizer. (a) TN (b) TP (c) TK (d) AHN (e) AP and (f) AK. Data are presented as the mean±SD (n = 6). Different letters (a, b and c) were statistically different ($p < 0.05$) according to Duncan’s new multivariate range test.

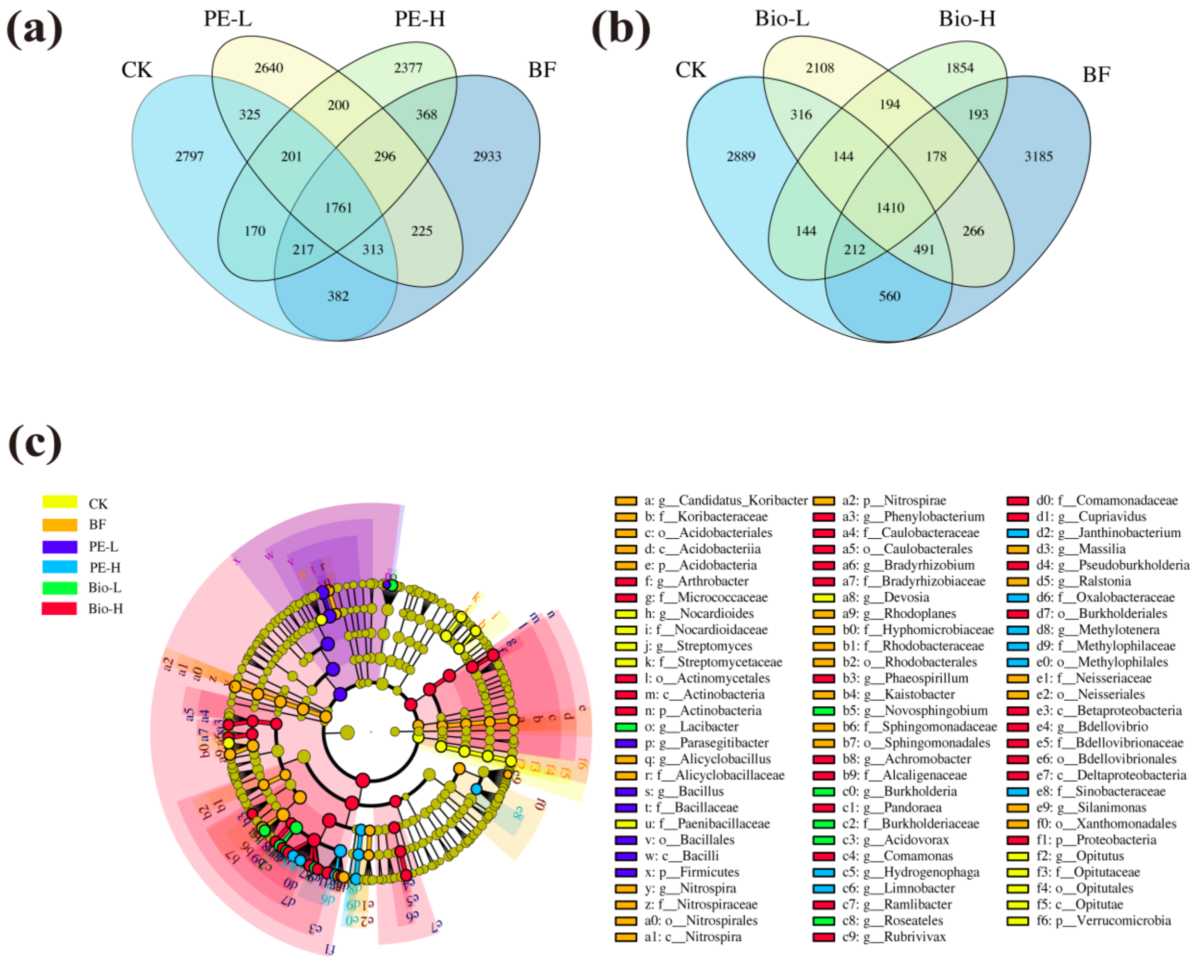

Figure S3. Effects of biofertilizers and MPs on rhizosphere bacterial community structure and composition. Venn diagram illustrating the number of (a) PE MPs and (b) Bio MPs OTUs in rhizosphere soil samples. (c) LEfSe analysis of bacteria in different treatment groups (LDA>3.5). Starting from the centre and moving outwards, each circle represents a different taxonomic level: domain, phylum, class, order, family, and genus. Taxa that exhibit significant differences are highlighted with colours.

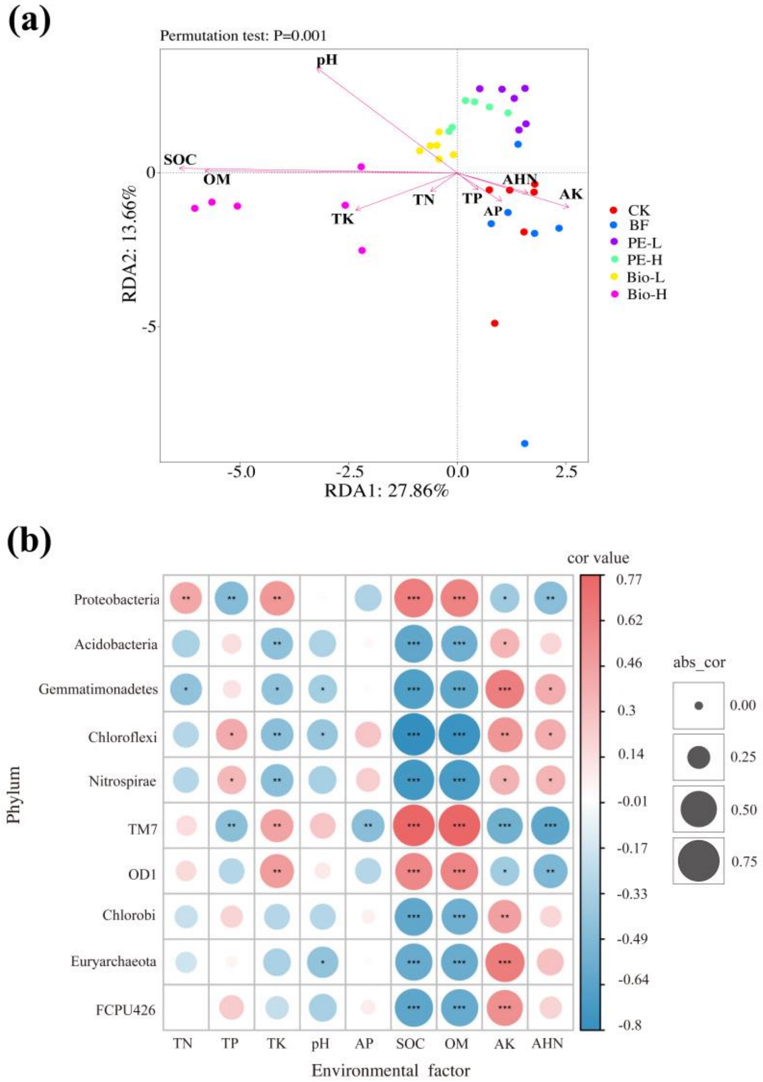

Figure S4. Correlation between rhizosphere soil bacterial communities and environmental factors. (a) RDA analysis between genus-level bacterial communities and environmental parameters. The red clippings indicate environmental parameters, and the length and direction of the arrows represent the degree of influence of soil physicochemical properties on the structure of bacterial communities and the positive and negative correlations between them. (b) Spearman's correlation analysis was conducted to examine the relationship between major bacterial communities and environmental parameters at the phylum level. (Statistical significance was denoted by *, **, and *** for p-values less than 0.05, 0.01, and 0.001, respectively)

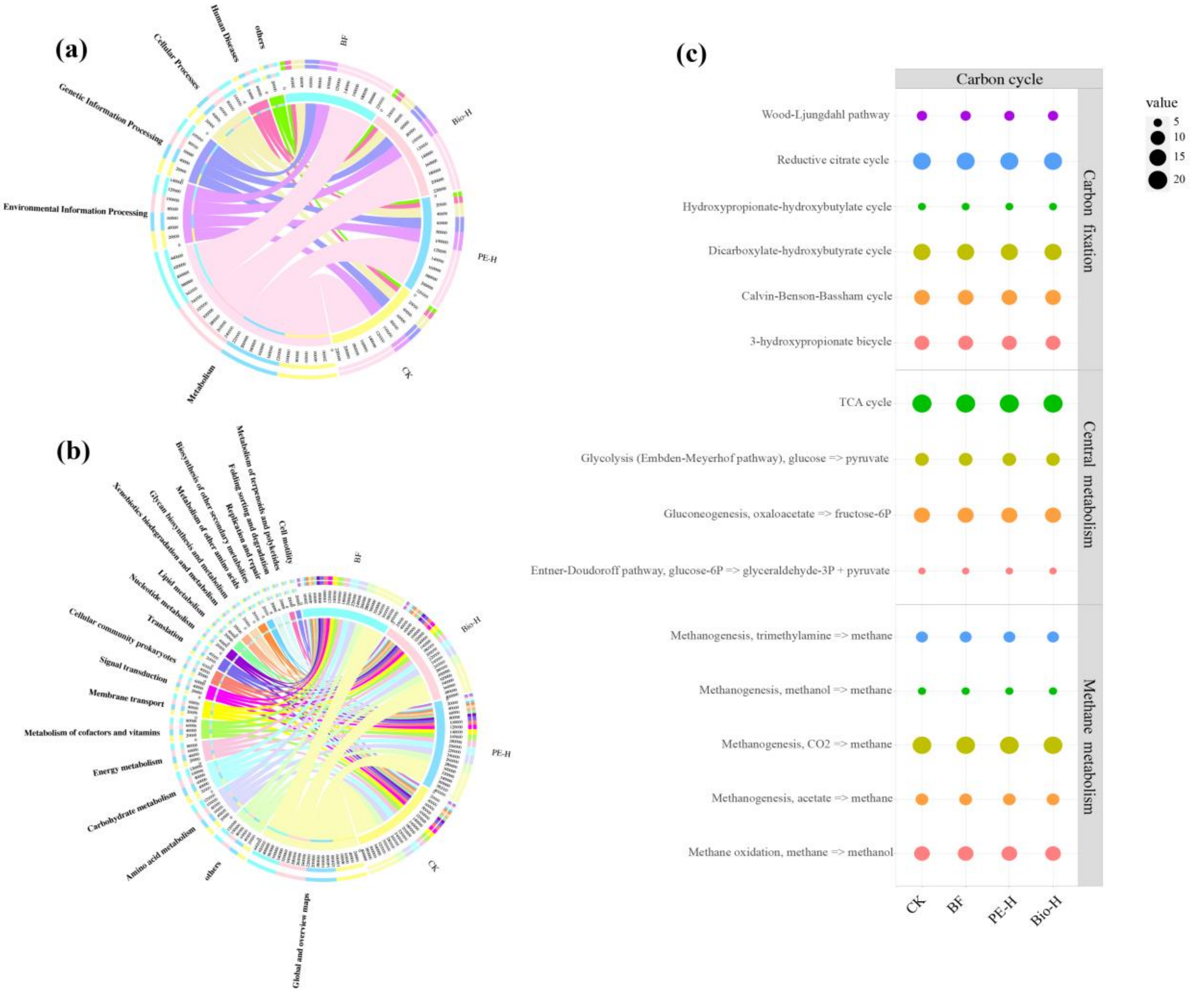

Figure S5. Functional composition of KEGG at primary (a) and secondary (b) levels. (c) Abundance bubble map of the carbon cycle pathwa

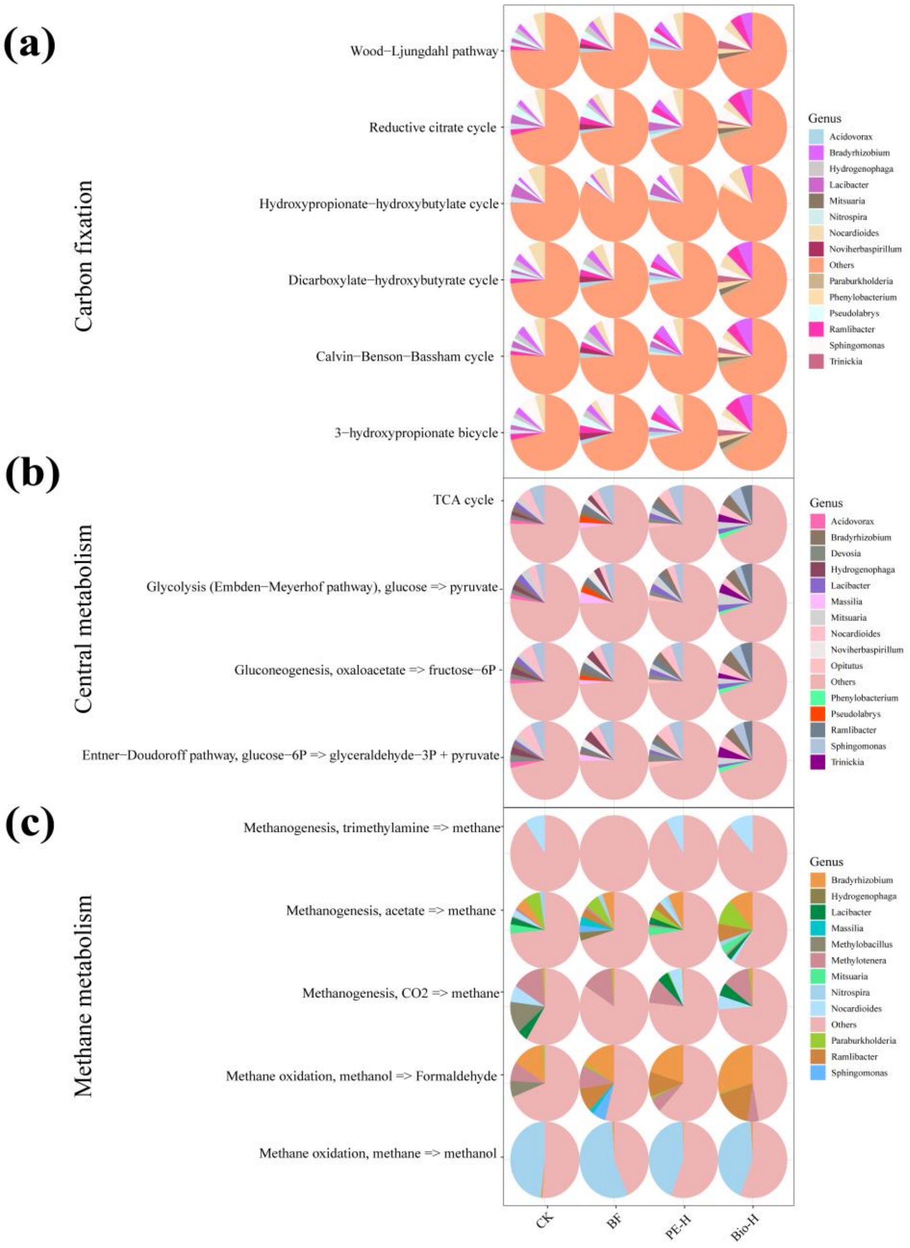

Figure S6. Abundance composition of genus level rhizosphere bacteria in (a) carbon fixation, (b) central metabolism, and (c) methane metabolism

Supplementary Tables

Table S1. Chemical and physical properties of soil sample (0 d incubation)

pH	OM ($g \cdot kg^{-1}$)	TN ($g \cdot kg^{-1}$)	TP ($g \cdot kg^{-1}$)	TK ($g \cdot kg^{-1}$)	AHN ($mg \cdot kg^{-1}$)	AP ($mg \cdot kg^{-1}$)	AK ($mg \cdot kg^{-1}$)
~6.00	~16.84	~0.84	~0.64	~14.84	~48.85	~178.20	~116.01

Note: Data are presented as the mean (n = 3).
